# Partial thrombosis of the corpus cavernosum – a malignancy mimicker

**DOI:** 10.1259/bjrcr.20210085

**Published:** 2021-09-10

**Authors:** Catarina Ala Baraças, Jorge Pinto, Maria Catarina Tavares

**Affiliations:** 1 Hospital Pedro Hispano, ULS, Matosinhos, Portugal

## Abstract

Partial thrombosis of the corpus cavernosum is a rare condition, typically seen in young patients. Etiology, physiopathology and treatment are still not entirely understood.

The authors report a case of a 49-year-old male with gastric cancer, who successfully treated a thrombosis of the corpus cavernosum conservatively. Diagnostic considerations and treatment options are discussed.

## Clinical presentation

A 49-year-old male, with mixed-type gastric adenocarcinoma, diagnosed 3 months before, developed perineal discomfort. Initial symptoms started when the patient stopped anticoagulation treatment. He was already submitted to chemotherapy and Roux-en-Y reconstruction after total gastrectomy.

The patient was initially diagnosed with acute epididymitis and started antibiotics.

The discomfort evolved to severe pain. Symptoms increased during urination. The patient denied hematuria, history of urinary tract infections, traumatic injury, drug abuse, or penile injection for erectile dysfunction treatment.

Although the pain resolved, the discomfort persisted, so the patient went to the hospital.

## Investigations/imaging findings

Physical examination revealed tenderness of the right portion of the perineum, along the ischiopubic ramus.

Laboratory analysis including white blood cell count, coagulation profile, C-reactive protein, and urinalysis were normal.

**Figure 1.**
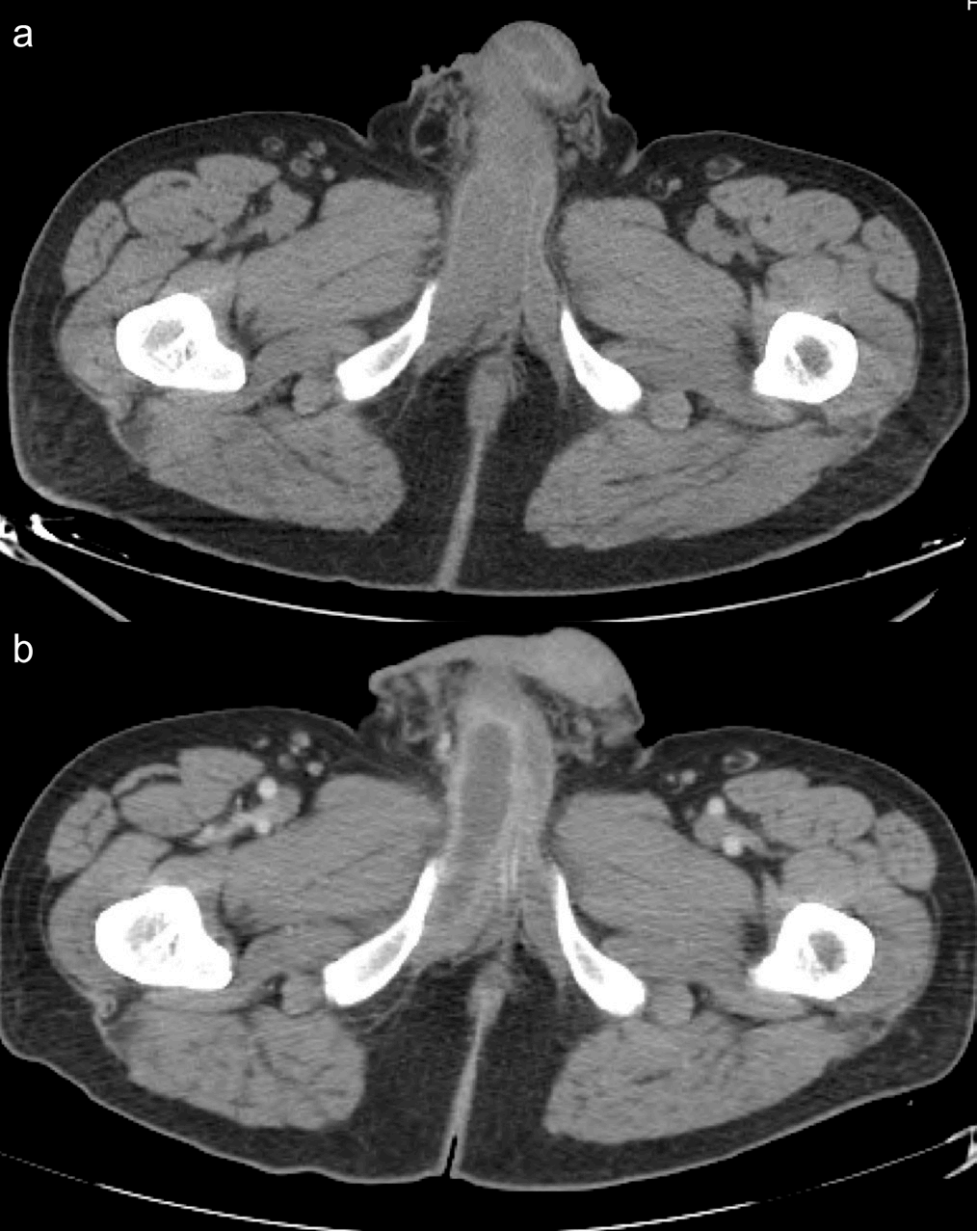
Non-contrast CT (1a) shows an asymmetrical enlargement of the proximal segment of the right corpus cavernosum. In contrast-enhanced CT (1b), the mass remains hypodense with a hyperdense rim of enhancement, causing mass effect on the left corpus cavernosum.

The patient performed an initial CT, before and after intravenous contrast injection (Figure 1), to study the cause of the pain and to rule out pelvic metastasis from gastric cancer.

An asymmetrical segmental enlargement with mass effect of the right corpus cavernosum was identified surrounded by an enhanced rim. Abdominal or pelvic metastases were excluded and no other masses or collections were founded.

To better characterize the right corpus cavernosum, additional Doppler ultrasound and MRI were performed.

**Figure 2.**
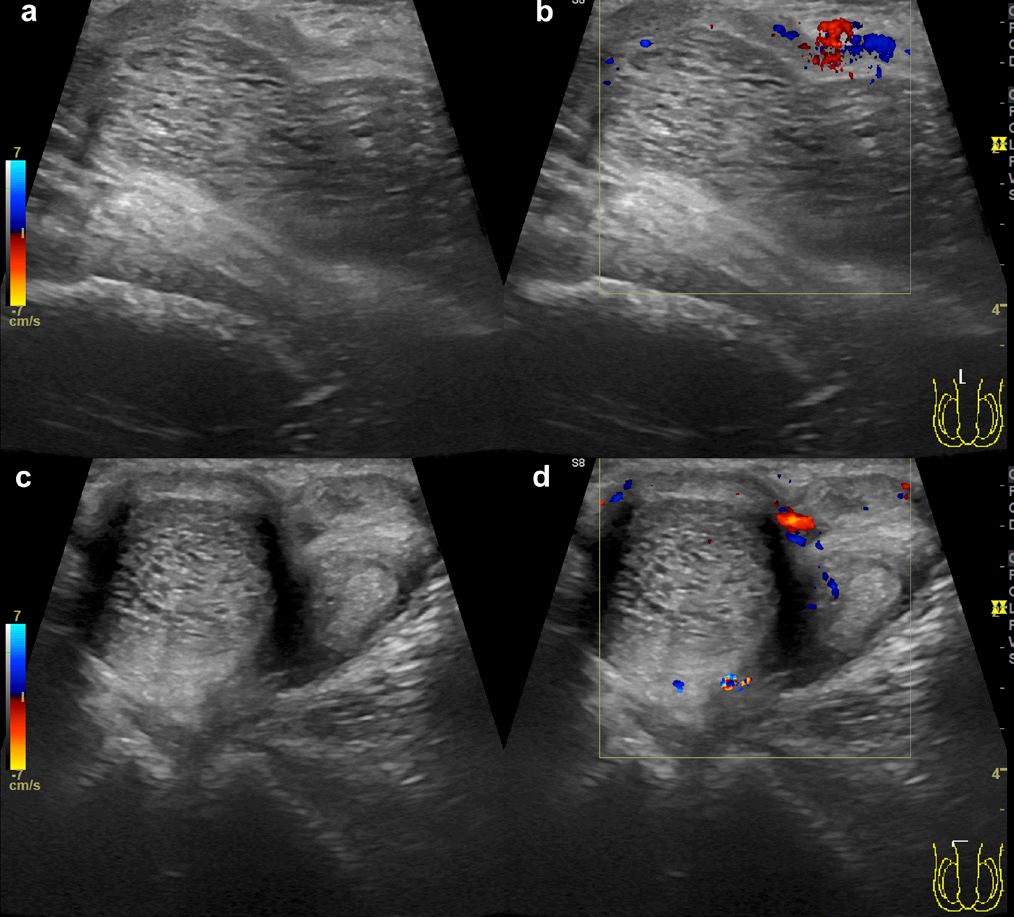
Ultrasound (2a, 2c) at the base level of the penis revealed a well-circumscribed right-sided corpus cavernosum, enlarged and round-shaped, with heterogeneous decreased echogenicity. Doppler ultrasound (2b, 2d) showed no vascularity inside the abnormal image.

Doppler ultrasound of the penis (Figure 2) showed expansion and heterogeneity of the proximal segment of the right corpus cavernosum with no flow inside. The left corpus cavernosum and the distal part of the right one appeared normal.

**Figure 3.**
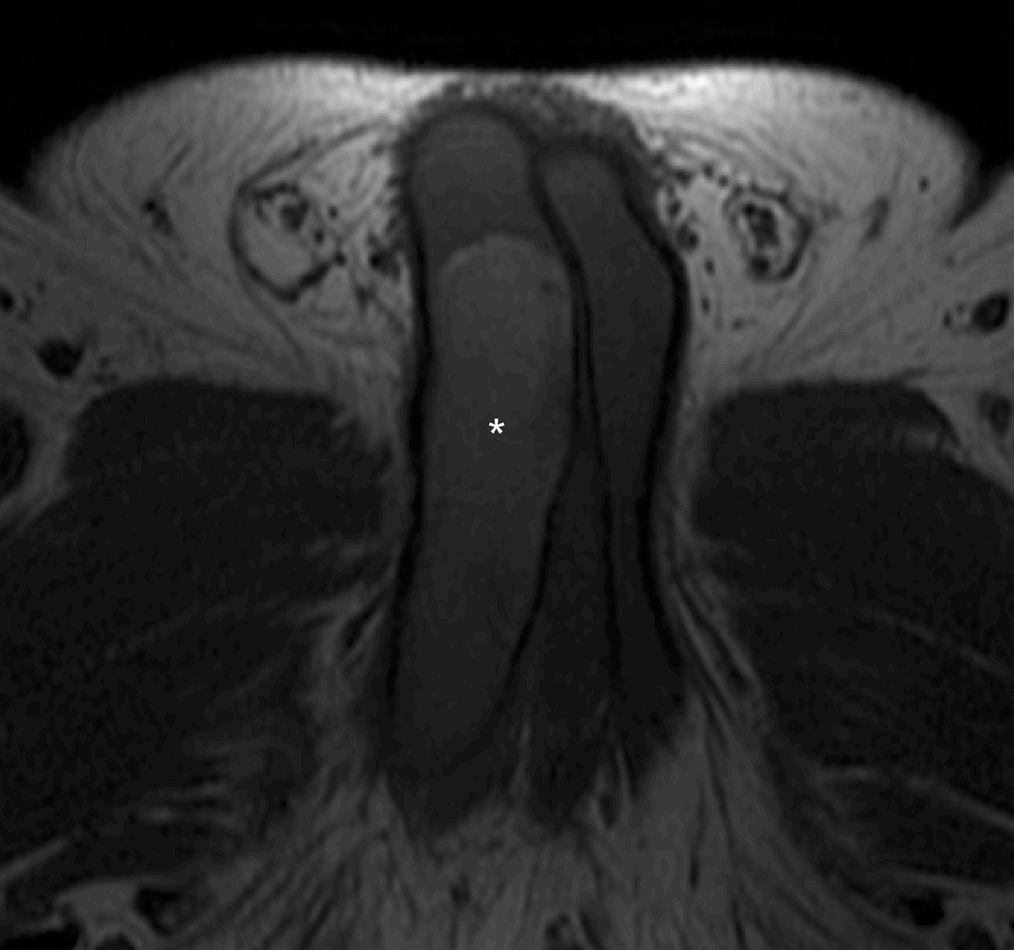
MRI (1.5 T) at the base of the penis. Axial view *T*_1_ weighted image shows the enlarged right corpus cavernosum, which is T1 hyperintense (*) comparing to the surrounding corpora. The left corpus cavernosum is displaced and compressed.

**Figure 4.**
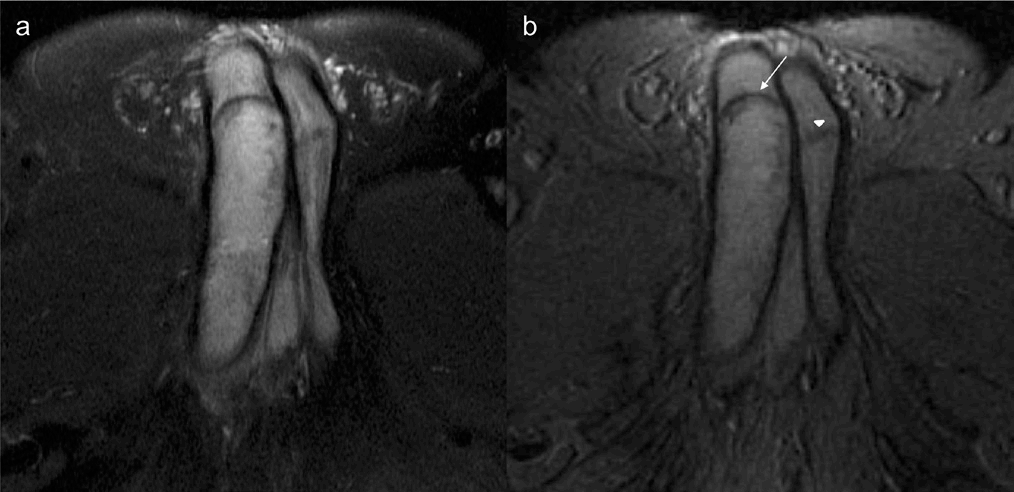
Axial *T*_2_ weighted FatSat (4a) and T2* images (4b) demonstrate iso/hyperintensity of the thrombus, due to extracellular methemoglobin. There is a membrane in the distal boundary of the thrombus displaying a low signal (arrow). There is a similar membrane in the contralateral corpus cavernosum (arrowhead).

**Figure 5.**
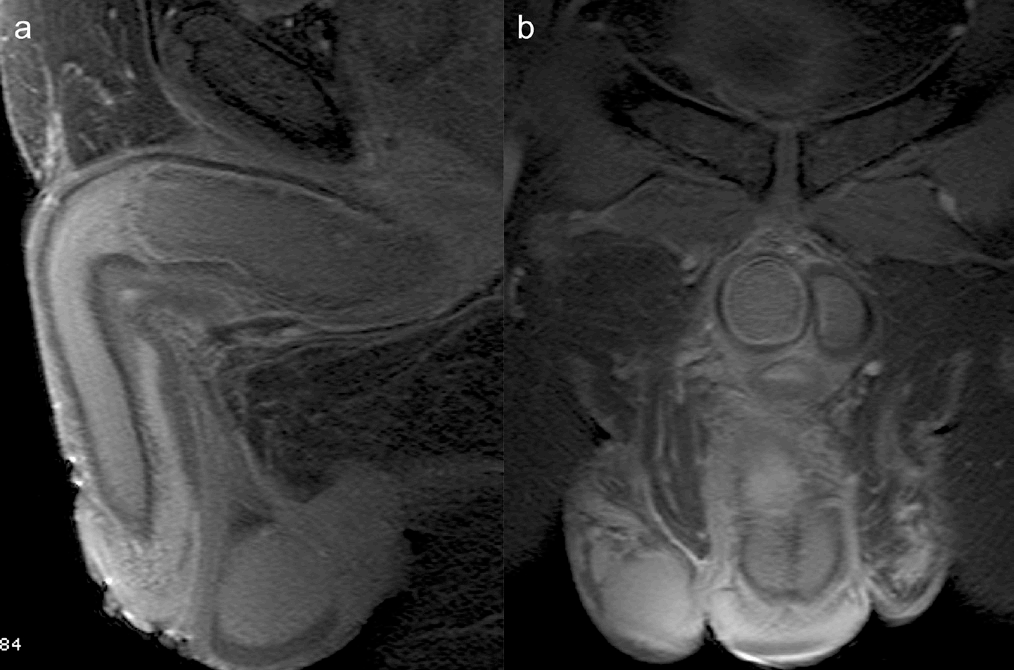
Sagittal (5a) and coronal (5b) *T*_1_ weighted images with gadolinium contrast-enhanced demonstrate a lack of enhancement of the thrombosed right-sided corpus cavernosum, with a rim enhancement.

Morphological MRI (1,5 T) of the penis, with pre and post-contrast gadolinium, was performed. *T*_1_ (Figure 3) and *T*_2_ weighted images (Figure 4) showed the increased signal intensity of the enlarged segment of the right corpus cavernosum, with the normal signal intensity of the remaining penis. After gadolinium administration (Figure 5), the normal portion of the penis showed regular enhancement. The enlarged proximal segment of the right corpus cavernosum showed peripheral enhancement with a lack of contrast uptake inside.

## Differential diagnosis

A subacute partial thrombosis of the right corpus cavernosum is the main differential diagnosis, according to the clinical history, laboratory findings, and imaging features. Adenocarcinoma is prone to produce procoagulant factors and both surgery and chemotherapy increase the risk of thrombosis. Implantation of a peripheral inserted central venous catheter (PICC tube) also increases the risk for thrombosis and thromboembolism due to direct damage of vascular endothelium.^1^

The absence of vascular flow inside the proximal portion of the right corpus cavernosum, with no fluid collections, combined with hyperintense T1, hyper/isointense T2 and T2* signal, and a peripheral rim of enhancement favor the diagnosis of a subacute thrombus in the corpus cavernosum.

Intracavernosal hematoma (in this case, a subacute hematoma) could also be a diagnosis to rule out, even though hematomas being usually caused by trauma. Spontaneous hematomas are rare. Furthermore, our patient stopped anticoagulation, which also favors thrombosis despite hematoma.^2^

Penile fracture with cavernosal rupture is a urological emergency and could be another diagnosis. However, it is clinically very often characterized by a history of penile trauma. The patient usually presents with a typically penile “eggplant deformity”. Ultrasound shows a hypoechoic tear in a normally hyperechoic tunica albuginea, associated with a collection or a hematoma. MRI is characterized by a hyperintense tear deformity on *T_2_* weighted sequences.^3^

Primary penile carcinoma is usually squamous cell carcinoma, and it usually appears in the penile gland. Primary penile cancers are typically hypointense on *T*_1_- and *T_2_* weighted images. The lesions usually have mild enhancement after gadolinium administration.^4^

Penile metastases usually present as multiple small lesions, diffusely distributed by the erectile tissue. They show T1 and T2 hypointensity compared to the normal corporal signal.^4^

Abscess of the corpus cavernosum is usually a complication after injection of intracavernous agents or an extension of another perineal abscess. It is clinically associated with penile cellulitis. A thickened fluid collection is seen on ultrasound examination. MRI shows a hyperintense T2 collection, with surrounding edema and cutaneous thickening.^5^

Fibrosis in the corpora cavernosa, namely Peyronie’s disease, results from repeated traumas. It is characterized by a clinically visible penile angulation, with palpable plaques, sometimes calcified, also seen on ultrasound and CT images. *T_2_* weighted images show linear intracavernosal low signal, due to fibrous plaques.^6^

## Treatment

The patient was treated with non-steroidal anti-inflammatory drugs and systemic anticoagulation. No surgical intervention was necessary.

Perineal examination revealed no palpable changes and imaging follow-up showed complete thrombus resolution. The patient remained asymptomatic.

## Outcome, follow-up and discussion

Partial thrombosis of the corpus cavernosum is a rare urological condition that occurs in males with a mean age of 30 years old.^7^

It is characterized by unilateral thrombosis of the proximal segment of the corpus cavernosum with distal corpus cavernosum and flaccid glans. Thrombosis of both corpora is even rarer.^8^

Etiology and pathophysiology persist unclear. Microtrauma to the corpora cavernosa is pointed as a possible mechanism.^7^ Traumatic sexual activity, cycling, malignancy, and hematologic diseases (sickle cell anemia and congenital spherocytosis) have been proposed as risk factors.^9–13^

CT scan can be more readily accessible in an acute situation. In a patient with malignancy, it allows excluding possible metastases.

MRI and Doppler ultrasound are often used for further differential diagnosis work-up.

Doppler ultrasound can demonstrate the flow pattern of the corpora cavernosa.

MRI is more helpful than CT in the diagnosis of penile thrombosis.^2,11^ MR imaging allows accurate tissue characterization and thrombus age. MRI can show different types of signals in *T*_1_- and *T*_2_ weighted images, depending on the stage of hemoglobin degradation. In this patient, images show the paramagnetic effect of methemoglobin degradation in the subacute phase of the thrombus.

Surgical reports refer to the presence of a fibrous septum, separating the proximal thrombosed portion from the distal flaccid segment.^10^ This thin membrane has been reported in several MR images.^8,11^ In our case, we can identify this thin membrane in both corpora cavernosa. It can represent an innate fibrous septum or a post-trauma sequela predisposing to developing thrombosis.

Treatment is almost always conservative. Nowadays, surgery is discouraged. It is only reserved for patients with symptoms and erectile dysfunction.^14^ Our patient became asymptomatic with conservative treatment.

## Learning points

- Partial thrombosis of the corpus cavernosum is a rare urological condition, usually in young males, clinically characterized by perineal pain or discomfort.
- Traumatic sexual activity, cycling, malignancy, and some hematologic disorders are believed to be predisposing factors.
- MRI is the most accurate imaging method to characterize tissue and determine the age of the thrombus. Subacute thrombus tends to be hyperintense on T1 and iso/hyperintense on *T*_2_ weighted imaging, due to methemoglobin degradation.
- CT and Doppler ultrasound are ancillary imaging methods to rule out differential diagnoses.
- Nowadays, conservatively treatment is more often preconized. Surgery is reserved for situations where the patient maintains symptoms.

## Patient consent

Written informed consent was obtained from the patient for publication of this case report, including accompanying images.
